# A Case of Fulminant Hepatitis due to Echovirus 9 in a Patient on Maintenance Rituximab Therapy for Follicular Lymphoma

**DOI:** 10.1155/2015/454890

**Published:** 2015-05-28

**Authors:** Ceri Morgan, S. J. Thomson, Joanne Legg, Santosh Narat

**Affiliations:** Worthing District General Hospital, Western Sussex Hospitals NHS Foundation Trust, Lyndhurst Road, Worthing BN11 2DH, UK

## Abstract

Rituximab is a CD20 monoclonal antibody commonly used in the treatment of haematological malignancies. It causes lymphopenia with subsequent compromised humoral immunity resulting in an increased risk of infection. A number of infections and viral reactivations have been described as complicating Rituximab therapy. We report an apparently unique case of echovirus 9 (an enterovirus) infection causing an acute hepatitis and significant morbidity in an adult patient on maintenance treatment of Rituximab for follicular lymphoma. We also describe potential missed opportunities to employ more robust screening for viral infections which may have prevented delays in the appropriate treatment and thus may have altered the patient's clinical course. We also make suggestions for lowering the threshold of viral testing in similar patients in the future.

## 1. Introduction

We report an apparently unique case of an echovirus 9 (an enterovirus) infection causing an acute hepatitis and significant morbidity in an adult patient on maintenance treatment of Rituximab for follicular lymphoma. Rituximab is a CD20 monoclonal antibody commonly used in the treatment of haematological malignancies [1]. It causes lymphopenia with subsequent compromised humoral immunity resulting in an increased risk of infection. A number of infections and viral reactivations have previously been documented to occur as a consequence of Rituximab treatment (including enterovirus meningoencephalitis and hepatitis B reactivation [2]) but no cases report an acute hepatitis as a direct consequence of enterovirus infection. We describe potential missed opportunities to employ more robust screening for viral infections which may have prevented delays in the appropriate treatment such as more timely use of immunoglobulin and Pleconaril or to possibly stop Rituximab maintenance steps which may have altered the patient's clinical course. We also make suggestions for lowering the threshold of viral testing in similar patients in the future.

## 2. Case Report

We describe an apparently unique case of echovirus 9 (an enterovirus) infection causing an acute hepatitis in an adult patient on maintenance treatment of Rituximab for follicular lymphoma. The risk of viral reactivation in patients on Rituximab treatment is well established with several reported cases of reactivation of John Cunningham virus (a virus implicated in progressive multifocal leukoencephalopathy in immunocompromised patients) and hepatitis B infection in particular [1]. Echovirus 18 infection has been described as causing an acute hepatitis in a non-Hodgkin's lymphoma patient after stem cell transplant [3] and Rituximab associated enterovirus infections have previously been reported causing both meningoencephalitis and myocarditis [2, 4]. However this appears to be the first case of fulminant hepatitis attributed to enterovirus infection causing significant morbidity in an adult patient on maintenance Rituximab therapy.

Our patient, a 70-year-old retired electrical engineer, fractured his hip after falling off his bicycle in February 2006. The X-ray of the hip showed an osteolytic lesion prompting a bone biopsy which demonstrated a low grade B cell lymphoma. A staging CT at the time stated there was no evidence of clinically significant lymphadenopathy and only moderate splenomegaly of 12.6 cm in diameter. The patient remained asymptomatic and a decision was made to “watch and wait.” Following an episode of left sided abdominal pain in January 2011, a CT demonstrated retroperitoneal lymphadenopathy and obstruction of the left ureter; there was also an increase in right axillary, mediastinal, left inguinal, and pelvic nodes. All other lymph nodes, specifically at the hepatic hilum, and spleen size remained stable. A lymph node biopsy from the left inguinal region demonstrated that the atypical lymphocytes were B cells (CD20 and CD79a positive). The atypical lymphocytes were also BCL2 and BCL6 positive and CD21 and CD23 positive highlighting expanded follicular dendritic cell meshwork consistent with low grade non-Hodgkin's follicular lymphoma. Treatment was commenced and the patient received a total of eight cycles of Rituximab and seven of CVP (cyclophosphamide, vincristine, and prednisolone). A posttreatment CT scan demonstrated a good partial response and he was therefore commenced on a two-year course of maintenance Rituximab on a three-month basis in the first instance in December 2011 at a dose of 750 mg, based on 375 mg per m^2^.

**Figure 1:**
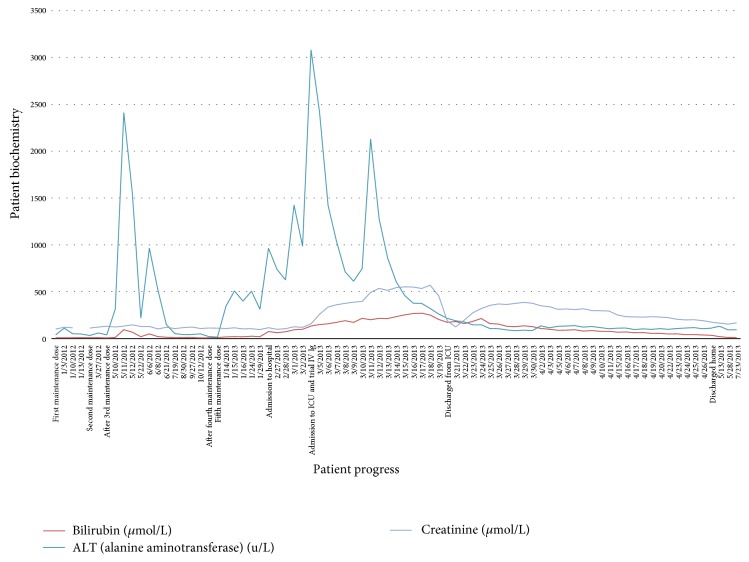
Summary of biochemistry findings: the graph shows the clinical events prior to and during the patient's admission to hospital and the pattern observed in both liver and renal function.

A routine liver function test (LFT) in June 2012 demonstrated total bilirubin of 50 *μ*mol/L (normal range <20 *μ*mol/L), alkaline phosphatase of 453 u/L (normal <130 u/L), and alanine aminotransferase (ALT) of 966 u/L (normal <40 u/L) (Figure 1). Rituximab was therefore suspended following which the LFT panel normalised. A subsequent CT demonstrated no disease recurrence and he was recommenced on bimonthly Rituximab in November 2012 at the previous dose of 750 mg. The patient attended hospital twice in the subsequent months with low grade fevers and malaise, once treated with Amoxicillin for a UTI and on the second occasion diagnosed with suspected bronchitis. His LFTs during these consults were mildly deranged and a further aetiological liver screen was ordered (anti-nuclear antibodies, anti-mitochondrial antibodies, anti-smooth muscle antibodies, liver and kidney microsomal antibodies, and anti-neutrophil cytoplasmic antibody) and viral serology was ordered where CMV IgG was positive. All remaining serologies including EBV and hepatitis A, hepatitis B, and hepatitis C were negative. A HIV test was also negative.

Further assessment was triggered in January 2013 by ongoing weight loss and malaise. LFTs were bilirubin 20 *μ*mol/L, ALP 543 u/L, and ALT 313 u/L and Rituximab was stopped. Shortly after this he presented with new onset confusion, fevers, and nausea. LFTs deteriorated again: bilirubin 75 *μ*mol/L, ALP 652 u/L, and ALT 964 u/L. He was therefore admitted by the Haematology Team for further management as an inpatient. Repeat serologies for hepatitis A, hepatitis B, and hepatitis C were all negative as was PCR for CMV. Similarly, EBV and HSV PCR were also negative. Immunoglobulin levels were IgG 3.27 g/L (6–16 g/L), IgM <0.25 g/L (0.5–1.9 g/L), and IgA 0.50 g/L (0.8–2.8 g/L); unfortunately the patient did not have immunoglobulin levels done at any time prior to Rituximab therapy.

The patient deteriorated quickly in hospital and was treated with broad spectrum antimicrobials (piperacillin/tazobactam and gentamicin) due to fevers (39.8°C) and features of sepsis. An abdominal CT reported acute acalculous cholecystitis and antibiotics were continued; however both liver and renal function continued to deteriorate. INR at this stage was 1.9, APTT 1.7, fibrinogen 1.5 mcg/mL, platelets 56, C-reactive protein 69 mg/L, and creatinine 259 *μ*mol/L.

The clinical picture progressed and he became more confused. In the context of acute jaundice and coagulopathy, concern was raised that this represented hepatic encephalopathy and, hence, acute liver failure. However, an arterial ammonia assay of 56.6 *μ*mol/L (1–50 *μ*mol/L) was not immediately supportive of that. Local hepatology opinion was sought and, due to his age, comorbidity, and apparent seronegative aetiology, he was not felt to be a transplant candidate. A plan was agreed to pursue supportive measures for his evolving multiorgan dysfunction and illness.

**Figure 2:**
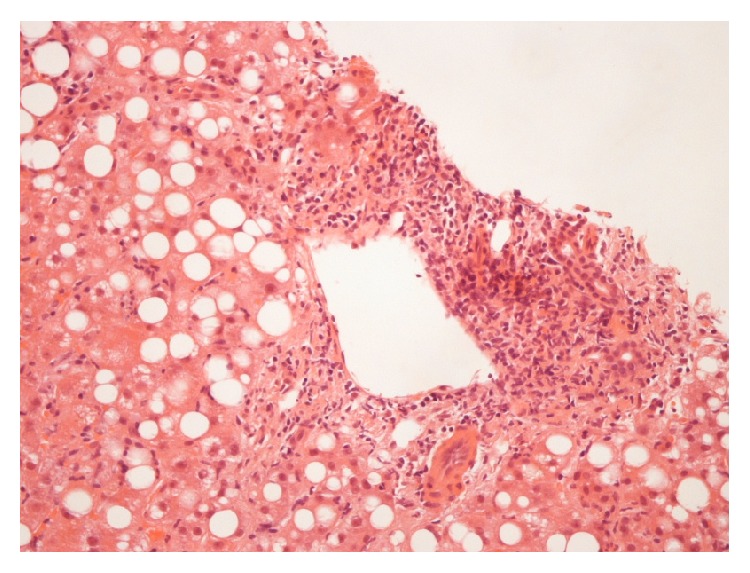
Histology findings: lymphocytic inflammatory infiltration of portal tracts, a finding suggestive of acute hepatitis.

Our patient continued spiking fevers of >38°C despite continued antibiotics and so an empirical trial of ganciclovir was commenced and a transjugular liver biopsy was performed. During this period, further real time viral PCR testing was performed on blood, and enterovirus RNA was detected at a “strong level.” This was later subspeciated as echovirus 9. Liver histology demonstrated predominantly lymphocytic inflammatory infiltration of the portal tracts with extension into the liver parenchyma and balloon degeneration of hepatocytes. There was also periodic acid-Schiff positive diastase resistant material in Kupffer cells. The report stated that this is a pattern described in disease states causing hepatocyte damage and necrosis. There was no evidence of cirrhosis, and the overall impression was that of an acute hepatitis (Figure 2). Echovirus 9 was also detected by PCR in the liver biopsy.

Our patient was subsequently admitted to ICU due to rapidly deteriorating renal function and difficulty in maintaining fluid balance on the ward. Prior to admission to ICU, his ALT rose to its highest value of 3082 u/L; he was jaundiced, bilirubin of 134 *μ*mol/L, and was hypoalbuminaemic, albumin of 22 g/L (35–50 g/L). A trial of intravenous immunoglobulin therapy (IV Ig) was commenced at 0.4 g/kg, a total dose of 2.4 g/kg being given. The authors are aware that this dose is slightly higher than the recommended total dose of 2.0 g/kg, but as the patient was in established renal failure requiring dialysis, it is not felt that this contributed to his renal failure. He was commenced on continuous venovenous haemofiltration due to progressive oligoanuric renal failure. His illness was further complicated by* Clostridium difficile* diarrhoea which was treated with oral vancomycin. He continued to suffer with agitation and confusion. An MRI brain reported mild inflammatory changes in the sphenoid sinus but no inflammatory changes in brain tissue. The possibility of an associated viral meningoencephalitis was postulated but a lumbar puncture was deferred due to coagulopathy and the multisystem nature of his condition.

He remained at the intensive care unit for a total of 17 days. Eventually renal and liver function was observed to improve and his requirements for organ support recovered. He was discharged to the ward where he completed a period of rehabilitation before being discharged. At follow-up, his memory has slowly improved although he does report being more forgetful. His follicular lymphoma remains in remission and he is not currently on any maintenance treatment. He is back playing golf twice a week but admits feeling more fatigued than he once did.

## 3. Discussion

Enteroviruses are single stranded positive sense RNA viruses that are transmitted via the faecal-oral route and have a peak transmission rate in the summer and autumn months. They are members of the Picornaviridae family and include the polioviruses, coxsackie A and coxsackie B, echoviruses, and the numbered enteroviruses. In immunocompromised adults, they are implicated in CNS disease [2] and they have been reported to cause sepsis and systemic infections in babies [5]. Treatment of enterovirus infections is reserved for encephalitis, myocarditis, neonatal infection, and B cell deficient hosts [6]. Previously the evidence for treating enteroviral, CNS infection in adults with immunoglobulin (IV Ig) alone in patients with compromised B cell response due to Rituximab maintenance is mixed, with one patient eventually dying after IV Ig of the infection [7] and another reporting a short lived improvement [1]. Our patient received IV Ig some two weeks into his stay and it is speculated that this may have been too late in affecting his clinical course. The drug Pleconaril which binds to the viral capsid and changes viral attachment and uncoating is unlicensed for use in enterovirus infection but has been used with some success in combination with IV Ig in CNS infection attributed to enterovirus infection in patients on maintenance Rituximab therapy [8].

The British Society of Haematology recommends that patients with follicular lymphoma have maintenance Rituximab after an appropriate induction regimen of chemotherapy [9]. The evidence base for this practice is robust with a recent meta-analysis demonstrating improved overall survival despite increased rates of infection in patients with relapsed follicular lymphoma but not untreated follicular lymphoma [10]. In individuals treated with Rituximab, it is their compromised humoral immunity that is thought to contribute to their vulnerability to viral infection. Rituximab is a chimeric monoclonal antibody directed against CD20 positive B cells. CD20 is expressed at all stages of B cell maturation with the exception of very early pre-B cell and mature plasma cell stages [11]. Rituximab causes profound B cell lymphopenia and can affect immunoglobulin levels up to 12 months after cessation of treatment [12]. An increase in grade 3 and grade 4 infections has been reported in meta-analysis of patients on maintenance Rituximab therapy but the rate of infections was not different whether it was given on 4-week, 6-week, 2-month, or 3-month basis [13].

There are several case reports of viral reactivation of hepatitis B and John Cunningham virus and resultant progressive multifocal leukoencephalopathy, the early features of which have some clinical resemblance to meningoencephalitis caused by enterovirus [2]. There are also reported cases of reactivation of CMV and infection of parvovirus B19, West Nile virus, and enterovirus as well as anecdotal cases of* Pneumocystis jirovecii*,* Mycobacterium*, and babesiosis [12, 13]. The clinical course and outcomes of these infections are often challenging as the compromised immunity leads to a more florid illness. This is also complicated by the low immunoglobulin levels present in some of these patients resulting in falsely negative serology testing, increasing our diagnostic dependence on more time consuming molecular techniques such as PCR. This may lead to delays in appropriate treatment.

In summary we have demonstrated a fulminant multisystem infection with severe hepatitis, features of liver failure, and associated acute renal failure due to echovirus 9 in direct relation to Rituximab therapy for a follicular lymphoma. In retrospect, the derangement in LFTs and febrile illness prior to his acute admission are likely to have been due to an evolving hepatitis and may have represented an opportunity for more robust screening for viral infection. It is also our impression that the associated features of confusion and disorientation may have been due to a coexistent meningoencephalitis for which enterovirus has also been historically implicated. A low threshold for comprehensive viral testing should be adopted in patients on Rituximab therapy who present with systemic symptoms, multisystem disorder, or more specifically abnormal liver function tests.
